# Reducing the infectious diseases burden through “life course approach vaccination” in India—a perspective

**DOI:** 10.3934/publichealth.2021045

**Published:** 2021-08-03

**Authors:** Sheikh Mohd Saleem, Sudip Bhattacharya

**Affiliations:** 1 Independent Public Health Researcher, Jammu &Kashmir, India; 2 Independent Public Health Researcher, Dehradun, Uttarakhand, India

**Keywords:** vaccine, life course approach, infectious disease, infections

## Abstract

The burden of vaccine-preventable diseases is increasing day by day across all age groups. However as per the universal immunization programme, we vaccinate only under-fives and antenatal mothers, a large portion of the vulnerable population remain unvaccinated and the concept of “life course approach” regarding vaccination is missing. It increases the overall burden to the already constrained Indian health care system. As India, now has become the largest manufacturer of multiple vaccines, we have continuous chain healthcare points (primary-tertiary level), with dedicated manpower in the public health sector that is why in this paper we are proposing to expand the horizon of the vaccination process using a “life course approach”. It will not only directly benefit the vulnerable populations (individual level), but also it can benefit the nation, indirectly. Although we may face challenges at multiple phases (conception to implementation), they can be overcome by multisectoral and multipronged innovations.

## 1. Introduction

A vaccine is a “product that stimulates a person's immune system to produce immunity/antibody to a specific disease and protecting the person from that disease.” The name of the process is called vaccination [1]. Vaccines are usually administered through multiple routes, like nasal, intradermal, intramuscular, and oral.

Historically, the first-ever successful vaccination was conducted by Edward Jenner in 1796 by use of cowpox serum to create immunity to smallpox patients [2]. Later, this practice gained widespread recognition and resulted in the eradication of a deadly disease like smallpox, globally. Up to the 1930s, vaccines and antitoxins were developed against deadly diseases like tetanus, diphtheria, cholera, anthrax, typhoid, plague, tuberculosis and many more. Till the last decade the vaccines were mostly used among the children for eradication of vaccine preventable diseases [3]. With increasing knowledge on the infectious agents, the extending benefits of vaccination from childhood throughout the entire lifespan is becoming popular and a new concept of “life course vaccination” came into existence. In simpler words, life course vaccination can be explained as by vaccinating and educating people about vaccination throughout their lives, a population with a better capacity to lead healthy, productive lives for longer can be build, which in turn will contribute to the sustainability of the healthcare systems and the productivity of the societies overall, for current and future generations [4]. In view of the emergence and re-emergence of infectious diseases and world's aging population, the necessity to invest in the preventive methods like life course vaccination is more crucial and important than investing in a curative care at present in global healthcare scenario [5].

In this paper, we highlight the benefits of vaccination beyond childhood i.e., life course vaccination, existing status of vaccination in India, possible solutions through life courses vaccination and its advantages over routine vaccination, challenges and recommendations that must be taken by policy makers, health care professionals and patients to ensure the full benefits from the strategy are retained.

## 2. Overall benefits of “life course approach vaccination”

As described in the Table 1, there are newer vaccines available which can benefit from at all age. Furthermore, there are changes to the body immune system which means that with advanced age the people are more prone to vaccine-preventable diseases. The reason being that they are less benefitted from the vaccines they have received during their childhood because of the need for booster doses of vaccines which they might not have received during the time over these years. Booster doses of vaccines provide additional protection against diseases like diphtheria, pertussis and tetanus if taken during the adolescent or adulthood period and thus may provide protection against disease for a longer period [6]–[8]. Currently, many vaccines are under developmental trials or have been launched against diseases like influenza, pneumococcus, zoster which have increased protection against vaccine-preventable diseases, especially among the elderly [9]. Although, studies have reported that those with chronic health conditions can have severe health outcomes but those who undergo vaccination even in adulthood can benefit themselves for example-the risk of cardiovascular events/complications are increased among those with chronic conditions who suffer from influenza but the vaccination against influenza almost reduces their risk to half [10]. Udell et al. [11] conducted a meta-analysis of 6 randomized controlled trials and assessed the benefits of the influenza vaccine in reducing cardiovascular events. The authors found that there was a lower risk of cardiovascular events than placebo or standard care without vaccination (2.9% vaccine vs. 4.7% placebo/control; relative risk: 0.64; 95% CI: 0.48 to 0.86; absolute risk difference: 1.74%; 95% CI: 0.81% to 2.67%; p = 0.003) [11].

The usefulness of influenza vaccines in the prevention of cardiovascular events and similar vaccines like hepatitis vaccines for prevention of hepatocellular carcinoma, HPV vaccine against cervical cancer have shown appreciating results worldwide and has many success stories [12]. From the above discussion, it is evident that vaccines are not only beneficial and helpful for the pediatric age groups but also the adult and the elderly and have been saving lives in this age groups also. According to the estimated, adult immunization save almost 2–3 million lives each year from infectious diseases worldwide [13].

Nevertheless, vaccines save lives, the data from the European countries showed that the median uptake among the nine countries was only 50.3% among those with certain chronic conditions [14]. Introducing the life course vaccination approach among the population will provide a direct benefit to the population in terms of cost-benefit and effectiveness ratio, as explained further. While such a long-term approach will help us to develop herd immunity among the population against certain vaccine-preventable disease which will be an indirect benefit of such an intervention in the long run like measles.

However, this 95% coverage of measles vaccination has been achieved in only 4 European nations and outbreaks due to gaps in vaccinations coverages at all ages with 53% of measles infection in 2017 have been reported among those who were above the age of 14 years [15]. Vaccines directly reduce the organisms and strains carrying resistant genes and indirectly via a secondary effect reduce the febrile illness that often leads to less use of antibiotics [16]. An indirect benefit of life-course vaccination is that it also significantly contributes to lowering antibiotic use in the community, thereby reducing antimicrobial resistance. A similar situation was seen in Ontario, Canada, when the vaccination against influenza was associated with a 64% reduction in the antibiotic prescription for respiratory infection among the local population among all ages [17]. Studies have also reported that vaccinations indirectly affect antimicrobial infections by preventing viral infections especially in the case of influenza vaccines which have been able to prevent secondary bacterial superinfections and antibiotic use by 13–64% [16]. If we consider similar success stories for hepatitis vaccines among children, studies have estimated that almost 7 million deaths have been averted only in the western pacific region with promising results in other regions of the world [18]. Furthermore, the researcher estimated that there is a 26% reduction in the number of cases and a 51% reduction in the number of deaths from cervical cancer among women who are vaccinated in their adolescent age against cervical cancers [19]. Most importantly, one among the most indirectly related benefits of life-course vaccination is the reduction in the estimated economic cost related to treating a disease which can be simply preventing with vaccination [20]. For example, between 2014 and 2019. Almost 1900 cases of measles were reported in the United States. The estimated cost to contain each case including the follow-up was $140,000, and the total cost associated with containing the spread of the outbreak was $266 million [21]. The United States spends $27 billion treating adults for the disease that could have been prevented with vaccination. Some models estimate that vaccinations administered to children born in the United States from 1994–2013 will prevent 322 million illnesses, 21 million hospitalizations, and 732,000 deaths throughout their lifetimes. The same cohort is predicted to have a net savings of $295 billion in direct costs and $1.38 trillion in total societal costs [22].

Population estimates in European countries suggest that an individual can spend up to €4000 to deliver up to 17 vaccines to complete vaccination across the life course approach which is quite affordable for them [23]. Another study reports that vaccination against up to 17 diseases throughout life in western European countries and full compliance with a national schedule can cost between €328 and €2,352 (vaccines costs only) and between €443 and €3,395 only [23].

**Table 1.** Few new vaccine preventable diseases and its burden in India.

Disease	Burden in India	Causative microorganism/vaccines available in India	Preferred group for vaccination	Mode of spread
Hepatitis B	India falls in the intermediate endemicity zone (prevalence of 2–7%, with an average of 4%), with a disease burden of about 50 million. In US, Hepatitis B-related liver disease kills about 5,000 people and costs $700 million annually (includes healthcare and productivity-related costs)	Hepatitis B virus/Hepatitis B vaccine (rDNA) pediatric & adult	For under 5 children: vaccination recommended in UIP programme throughout the country For other age groups: recommended for those with liver cirrhosis/hepatoma cellular carcinoma on recommendation from treating physicians	Blood borne, Vertical transmission
Cervical cancer	In India, cervical cancer contributes to approximately 6–29% of all cancers in women. The age-adjusted incidence rate of cervical cancer varies widely among registries; highest is 23.07/100,000 in Mizoram state and the lowest is 4.91/100,000 in Dibrugarh district. In US, over 6 million new infections annually; HPV strains cause 70 percent of all cervical cancers, and also cause cancer of the anus, penis, mouth, and throat	Human papilloma virus/there are two types of HPV vaccines available in India. Both of them are licensed globally. The first one is a quadrivalent vaccine called Gardasil, while the second is a bivalent one by the name cervarix.	Adolescents' girls but not mandatory in India	Sexual route
Influenza	A total of 127 092 (95% CI = 64 046-190 139) influenza-associated respiratory and circulatory deaths across all age groups occur annually in India In US, approximately, 3,300 to 49,000 deaths and more than 200,000 hospitalizations annually; over $10 billion in costs associated with a moderately severe seasonal outbreak	Influenza Virus/Trivalent-Quadri-valent Influenza vaccines	Recommended for all Age groups	Droplet Infection
Dengue	The overall seroprevalence of DENV infection in India was 48.7% (95% CI 43.5–54.0), increasing from 28.3% (21.5–36.2) among children aged 5–8 years to 41.0% (32.4–50.1) among children aged 9–17 years and 56.2% (49.0–63.1) among individuals aged between 18–45 years. The seroprevalence was high in the southern (76.9% [69.1–83.2]), western (62.3% [55.3–68.8]), and northern (60.3% [49.3–70.5]) regions. The estimated number of primary DENV infections with the constant force of infection model was 12,991,357 (12,825,128–13,130,258) and for the age-dependent force of infection model was 8,655,425 (7,243,630–9,545,052) among individuals aged 5–45 years from 30 Indian states in 2017.	Dengvaxia® Vaccine	Recommended Age 9–45 years old	Spread to people through the bites of infected Aedes species mosquitoe
COVID-19	Current COVID-19 cases as of 6^th^ May 2021 21,255,125, deaths: 231,542	Covishield® (AstraZeneca's vaccine manufactured by Serum Institute of India) and Covaxin® (manufactured by Bharat Biotech Limited).	Recommended for people aged 18 and above Only, excluding pregnant & lactating women	Droplet infection, Direct contact, Indirect mode of spread

The immunization programme in India was introduced in 1978 as the “Expanded Programme of Immunization” (EPI) by the Ministry of Health and Family Welfare, Government of India. Later in 1985, the programme was modified as the ‘Universal Immunization Programme’ (UIP) which was implemented in a phased manner to cover all the districts in the country [24],[25]. UIP is one of the largest public health programmes targeting close to 2.7 crore newborns and 2.9 crore pregnant women annually [24],[25]. Under the UIP programme, immunization is providing free of cost against 12 vaccine-preventable diseases, especially to under-five children and pregnant women.

In India, like other countries of Europe, the focus of immunization has mainly been on the pediatric group and child-bearing women only because the rate of mortality is the more in this age group and interventions due to immunization save millions of children every year. Moreover, childhood mortality is generally regarded as an important national indicator of the health care system as well as socio-economic development.

We understand that vaccination in children is essential, but we should not limit ourselves there only, this is because, at present newer infectious diseases like bird flu, swine flu, COVID-19 are emerging at a rapid pace and affecting all ages across the globe [26]–[27]. Along with these emerging and re-emerging infectious diseases, the incidence and prevalence of cervical cancers (by HPV infection), cirrhosis of the liver (Hep-B infection), and other vaccine-preventable disease are increasing. As we try to discuss the burden of various disease which can be prevented through efforts of vaccination, we must realize that the burden of Hepatitis B in India is about 2–7% with an average of 4% [16]–[20].

Similarly, cervical cancers are among the leading causes of cancers among women in India accounting for approximately 6–29% of all cancers in women [28]. On the other hand, the annual influenza-associated respiratory mortality rates were found to be highest for those ≥65 years (51.1, 95% confidence interval (CI) = 9.2–93.0 deaths/100,000 population) followed by those <5 years (9.8, 95% CI = 0–21.8/100,000) reported from India [29]. Similar seroprevalence studies also reported the burden of dengue from India [30]. It was reported that the seroprevalence of DENV infection in India was 48.7% (95% CI 43.5–54.0), increasing from 28.3% (21.5–36.2) among children aged 5–8 years to 41.0% (32.4–50.1) among children aged 9–17 years and 56.2% (49.0–63.1) among individuals aged between 18–45 years [30].

On the other hand, in two randomized trials, the efficacy of the human papillomavirus vaccine was assessed among women aged between 15 to 25 years of age, it was concluded that the human papilloma vaccine was more efficient to prevent cervical cancer lesions caused by HPV [27]. Similar evidence reports that flu vaccines saved almost 40,000 lives over 9 years in the United States only [2005 to 2013–14] [31].

A study from China on the cost-effectiveness of the single dose of booster among 10-year children made a significant difference in term of cost perspective. Costs associated with the management of chronic hepatitis emerged as the most influential parameters in the sensitivity parameters. However, it was observed that the cost-effectiveness ratios remained below the GDP levels in China in the “worst-case” scenarios even when explored in the sensitivity analysis scale [32].

In a quest to achieve the full potential, improve quality of life and reduce morbidity and mortality, life course approach vaccination may prove quite useful [33]. We believe that the Indian public health system is currently reached a stage where the basic infrastructure for UIP, vaccine manufacturing and production, cold chain information management system, a system for vaccine delivery, and capacity building are at an optimum place with more than 145,894 subcenters, 23,392 primary health centres, 4808 community health centres, Approximately 500 Private medical Colleges, 20 Apex Institutions of Medical Repute and more than 700 District Hospitals and Government Medical Colleges respectively [34]. A potential to expand the immunization programme beyond usual pediatric and childbearing women vaccination activities exists to include vaccinations for adolescents, adults, and elderly depending on disease burden in a geographical area and cost-effectiveness of the intervention [35]. Here the concept of life course approach (covering all age groups, considering different settings) comes into play where we propose that all under-five children should be vaccinated either at the Anganwadi's centres or the primary health care centres, school-going children can be vaccinated at the schools only, college-going students at the college. Now, for adults, there will be two groups, office going group/organized sector may get vaccination through their respective offices while those in non-office groups/unorganized sector can get vaccinated at NGOs or primary health care centres, geriatrics can also get vaccinated at old age homes, NGOs or PHCs. Pregnant women already register a beneficiary under UIP and can avail the services at ANC clinics as a rule (Table 2).

**Table 2.** Proposed “life course vaccination” approach.

Additional vaccines	Can be given to whom	At what place	By whom
Hepatitis B vaccine	Adults Geriatrics	Offices, NGOs, PHCs, Old Age Homes	Health Officers, Community Health Workers, volunteers, Public Health Workers
Human Papilloma Vaccine	Adolescent Girls	Schools and Colleges,	School Health Nurses, Health & Wellness ambassadors
COVID-19 vaccine	Adults & Geriatrics	Offices, NGOs, PHCs, Old Age Homes	Health Officers, Community Health Workers, volunteers, Public Health Workers
Influenza vaccine	Children Adults & Geriatrics	Offices, NGOs, PHCs, Old Age Homes, School and Colleges, Anganwadi centers etc	Health Officers, Community Health Workers, volunteers, Public Health Workers

## 3. Challenges ahead

Among the developing nations, India is doing good in pediatric vaccination program and it's a successful public health intervention so far. In regard to the adult vaccination, even to the published guidelines from the World Health organization (WHO), there is a lack of consensus by the National organizations regarding the strategies for adult vaccination. In response to this, the Association of Physicians of India (API) decided to fill this void regarding technical guidance for adult immunization strategies in India. The API observed that the surveillance data regarding the burden of infectious diseases from India is lacking. These recommendations equipped the physicians with data on vaccines, including dosage, indications, delivery frequency and administration [36].

Furthermore, the major challenge what drives people against the vaccination is their perception and attitude towards it. Many barriers which can be addressed include organizational barriers, patient related barriers (inadequate knowledge and awareness, negative perception and attitude, low health literacy), health care system barriers (lack of prioritization of vaccine, lack of recall system and delivery, lack of monitoring and data, lack of funding), health care provider related barriers (inadequate knowledge and awareness, negative perception and attitude, lack of effective communication with patient), and socioeconomic factors ( lack of inaccurate information in the public media, anti-vaccine movements, denial of disease risks, overconfidence vaccine paradox) [37]–[40]. However, when a country like India will decide to go for adult life course vaccination, it would face challenges like the way it faced while vaccination people against COVID-19. The challenges such as logistics of dispensing the vaccine to the vast population may be a big challenge, managing cold chain points which are currently just 30,000 in the country need to be augmented, managing the adequate vaccine supplies and stock, managing health care providers for vaccination booths for adult vaccination and also deciding which vaccines to be included in life course vaccination schedule and can Government afford to provide them free of cost to their people, that's the big challenge [41].

It is not such efforts were not made to set up dedicated adult vaccination centers in the country like India. One such example is from a premier medical institute like All India Institute of Medical Sciences (AIIMS), Jodhpur when in the year 2018 a total of 1,388 individuals attended the adult vaccination clinic. Majority (42%) received TT vaccines followed by (20%) individuals who received post-exposure prophylaxis for dog bites. Mere (15%) individuals got pre-exposure shots for yellow fever vaccines, (8%) for hepatitis B, (7%) for pneumococcal, (3%) for typhoid and only (1%) against influenza respectively [42]. The data from premier medical institute like AIIMS Jodhpur clearly indicates that even with the availability of adult vaccination services, people are not effectively utilizing such services which point towards decrease knowledge, information, and inadequate advocacy at various levels.

The prime challenge before us is the perception of the public for the adult vaccination campaigns if at all this is going to take place. Vaccine hesitancy has been recognized as one of the top ten public health threats all over the world [43]. As an example, India reported 12–70% vaccine hesitancy for polio vaccine during the early phase of the campaign [44].

Secondly, the surveillance and vaccination data/system for immunization beyond childhood age is presently nonexistent in the public health sector and need to be developed. An adequate initial investment in the surveillance system and collection of data through integrated disease surveillance programme (IDSP) and additional data collection from the already available maternal child health (MCH) cards must be a priority. We do acknowledge that the initial investment will be huge in terms of money, materials, and manpower but in a long run it will give long term dividend in the form of decrease in morbidity and mortality and improvement in the health indicators of India.

## 4. Conclusions and recommendations

Immunization recommendations are wide and are not limited to child immunization in countries like Poland, Romania, and Bulgaria like other parts of the world where life-course vaccination had been given a priority especially among the healthcare workers and elderly. In our opinion, the world is witness to the many epidemics and pandemics to date (Spanish flu 1918, Influence 2009, COVID-19 2019) and a threat of emerging and re-emerging diseases is quite real in the near future [44]. India, is going to house the largest populous country of the world in coming years and no doubt the burden of the vaccine-preventable disease will be immense [45]. This seems the right time to allocate resources to set future priorities to find significant gaps in the uptake of vaccination beyond childhood. Furthermore, a consensus must be agreed upon by the policymakers, civil society groups, non-political organizations and this life course vaccination approach must embed with the current UPI programme irrespective of income level. For the life course vaccination approach, we may perceive the shortage of skilled manpower. For this, we need to fill this gap by integrating community-based healthcare professionals, such as a pharmacist, chemists, regarding vaccine delivery. Moreover, vaccination may be given in non-health settings for example school-going children will be vaccinated in schools, adolescents in colleges, the working class in offices, non-working class in primary health centres, under-five children, pregnant women in primary health centres. The Elderly could either take vaccinations in old age homes, NGOs, or primary care centres (Table 2). This seems the right time to adopt a setting-based life course approach for those who are at risk with the right vaccine, at the right time and right place regardless of their age which would eventually improve their health status and quality of life. Ultimately the nation will become more resilient for vaccine-preventable diseases.
